# Transcatheter aortic valve replacement in patients with aortic stenosis and cardiac amyloidosis

**DOI:** 10.1016/j.ijcha.2022.101008

**Published:** 2022-03-21

**Authors:** Tasveer Khawaja, Rahul Jaswaney, Shilpkumar Arora, Akhil Jain, Nirav Arora, Luis Augusto Palma Dallan, Sunghan Yoon, Mohammed Najeeb Osman, Steven J. Filby, Guilherme F. Attizzani

**Affiliations:** a Department of Internal Medicine, Case Western Reserve University, University Hospitals, Cleveland, OH, United States; b Department of Cardiology, Case Western Reserve University, University Hospitals, Cleveland, OH, United States; c Department of Internal Medicine, Mercy Catholic Medical Center, Darby, PA, United States; d Lamar University, United States

**Keywords:** Aortic stenosis, Cardiac amyloidosis, TAVR, Heart failure

## Abstract

**Background:**

Though the co-prevalence of aortic stenosis (AS) and cardiac amyloidosis (CA) is increasingly recognized, the role of transcatheter aortic valve replacement (TAVR) in patients with CA remains unclear.

**Methods:**

The National Readmission Dataset (2016–18) and ICD-10 codes were used to identify those with CA and AS, in conjunction with TAVR status. The primary outcome was a composite of heart failure (HF) readmissions and all-cause mortality. All outcomes were followed up to 1-year with a median follow up time 172-days. Kaplan-Meier curves and multivariate cox-proportional hazard regression were used for time-to-event analysis.

**Results:**

Of 1,127 CA patients, 92 (8.2%) had undergone TAVR. Patients with CA who received TAVR were younger and more commonly had coronary artery disease (67.3% vs 44.2%). Teaching (93.6% vs 81.1%) and large hospitals (77.7% vs 59.3%) performed more TAVRs. In multivariate analysis, TAVR was associated with an improved primary outcome (8.9% vs 24.4%, HR:0.32; 95% CI 0.14–0.71, p = 0.007) and with reduced HF readmissions (3.8% vs 19.4%, HR:0.22; 95% CI 0.07–0.68, p = 0.008). All-cause mortality was numerically lower in TAVR patients with CA but did not reach statistical significance.

**Conclusions:**

CA patients who receive TAVR are younger, and the procedure is more commonly performed at large, teaching hospitals. TAVR was associated with a lower primary composite outcome of HF readmissions and all-cause mortality.

## 1 Introduction

Cardiac amyloidosis (CA) is recognized as a heterogeneous disease of abnormal protein deposition in the heart and has been associated with a growing number of cardiovascular conditions, including heart failure, arrhythmias, and valvular disease [1]. The interaction between aortic stenosis (AS) and CA is of growing interest due to the frequency with which these pathologies have been noted to coexist [1]. Conservative estimates of the prevalence of the acquired transthyretin (ATTR) subtype of CA in patients with AS have ranged from 4% to 29% [1]. As this overlap in pathologies continues to be explored, important questions regarding the optimal management strategy in this cohort must be answered. The mortality rate of patients with all forms of CA is considerable. The median survival of patients with immunoglobulin light chain (AL) CA has been estimated at 6 months from the onset of heart failure symptoms [2]. Median survival is higher in those with ATTRm CA and ATTRwt CA; but in both groups, survival is less than 4 years from diagnosis [3]. Given this data, concerns have been raised about the futility of aortic valve replacement (AVR) in patients with CA [3]. Thus, the objective of this study was to determine practice patterns and outcomes of TAVR in patients with AS and CA [4].

## 2 Methods

Data was extracted from the 2016–2018 Nationwide readmission database (NRD). NRD is a subset of the Healthcare Cost and Utilization Project (HCUP), sponsored by the Agency for Healthcare Research and Quality. NRD from 2016–18 contains data from approximately 17 million discharges, across 28 geographically dispersed states. This dataset accounts for 60% of the total U.S resident population and 58.2% of all U.S. hospitalizations [5]. NRD has been studied and validated in multiple previous studies [6], [7]. Due to the use of de-identified patients, institutional review board approval was not required.

**Fig. 1:**
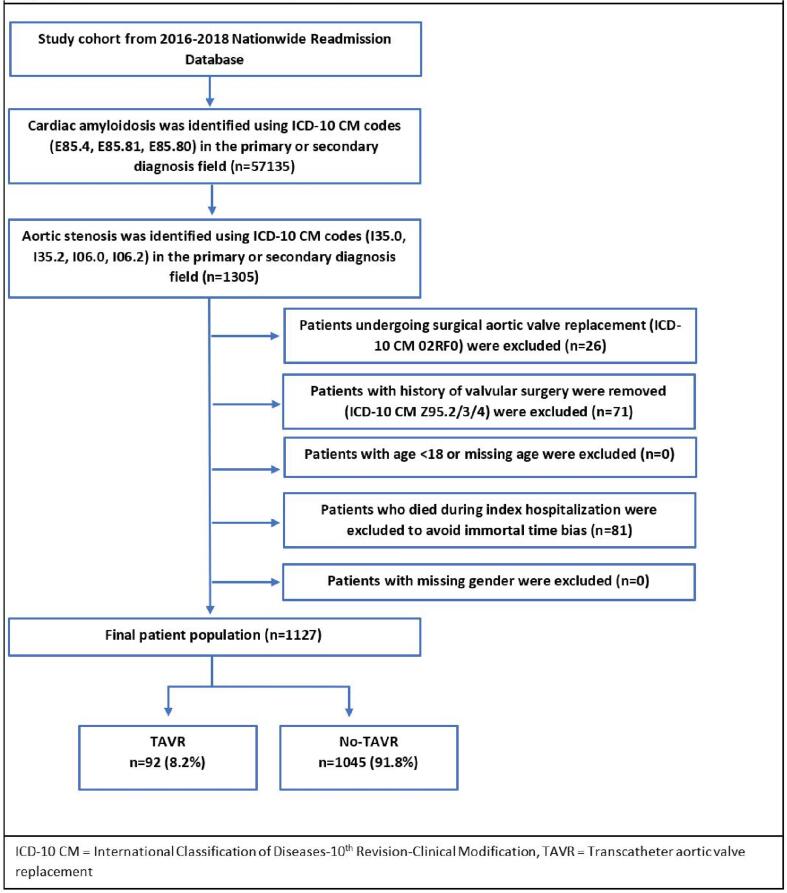
Patient Selection and Study Design.

We identified patients with cardiac amyloid using previously described ICD-10 CM codes (E85.4, E85.80, E85.81) in the primary or secondary diagnosis fields [8]. Aortic stenosis was identified using ICD-10 CM codes (I35.0, I35.2, I06.0 and I06.2) in either the primary or secondary diagnosis fields. Patients undergoing surgical aortic valve replacement (ICD-10 PCS: 02RF0) and those with a history of heart valve replacement (ICD-10 CM codes: Z95.2, Z95.3, Z95.4) were removed. Patients with missing information on age, gender, mortality, or age < 18 were removed. Patients who died during the index hospitalization were excluded to avoid the immortal time bias (Fig. 1).

We used the variables provided in the NRD by HCUP to identify baseline characteristics including age and gender, hospital characteristics such as bed size and teaching status, and other patient-specific aspects including primary payer, admission type, and admission day of the week [9], [10]. We utilized the ICD-10-CM codes provided by the Elixhauser comorbidity index calculator given by HCUP to identify obesity, hypertension, diabetes, chronic obstructive pulmonary disease (COPD), alcohol disorder, peripheral vascular disease, and anemia [11], [12] (**Supplementary Table 2**). Other comorbidities such as obstructive sleep apnea (OSA), prior coronary artery disease (CAD), family history of CAD, chronic kidney disease stage 3 or more (CKD), prior coronary artery bypass grafting (CABG), prior percutaneous coronary intervention (PCI), hyperlipidemia, previous stroke/transient ischemic attack, tobacco use, and atrial fibrillation (AF) use were identified using appropriate ICD-10-CM codes (**Supplementary Table 2**). Hospital size was determined by number of beds and categorized so that approximately one-third of the hospitals in each region, location, and teaching status combination would fall within each bed size category [13].

The intervention of interest was TAVR. TAVR was identified using ICD-19 PCS codes of 02RF38H, 02RF38Z, 02RF3KH, 02RF3KZ in either the primary or secondary procedure codes. The primary outcome was a composite of all-cause mortality and heart failure readmissions. All-cause mortality was provided by HCUP. Any patients with a primary readmission diagnosis of heart failure (ICD-10 CM codes: I11.0, I13.0, I13.2, I50, E85.4, E85.82, I35.0 and I35.2), aortic stenosis (ICD-10 CM codes: I35.0, I35.2, I06.0 and I06.2), or amyloidosis (ICD-10 CM codes: E85.4, E85.81, and E85.80) was considered a heart failure readmission.

SAS 9.4 (SAS Institute Inc., Cary, North Carolina) was used for statistical analysis. Categorical variables were compared using the Chi-Square test, and continuous variables were compared using the Student’s *t*-test (Table 1). A two-tailed p-value of 0.05 was designated as statistically significant. Time to event analysis was utilized. Cumulative event rate was generated using Kaplan Meier curves. We ran univariate and multivariate cox-proportional regression models for individual outcomes. We adhered to methodological standards of HCUP. All hazard ratios were adjusted for age and gender.

**Table 1:** Baseline Characteristics.

	No TAVR (%)	TAVR (%)	Overall (%)	p-value*
Patient populations	91.80	8.20		
Age (Years)				0.063
≤ 80	41.26	51.19	42.08	
>80	58.74	48.81	57.92	
Gender				0.574
Male	62.03	64.99	62.28	
Female	37.97	35.01	37.72	
Comorbidities^‡^				
OSA	12.07	13.61	12.20	0.664
Obesity	8.61	6.30	8.42	0.443
Hypertension	88.50	88.18	88.47	0.925
Diabetes	27.09	26.56	27.05	0.911
History of TIA or Stroke	25.72	14.12	24.77	**0.013**
COPD	16.26	17.99	16.40	0.666
CKD stage 3 or more	52.42	52.43	52.42	0.998
Prior CABG	7.23	7.85	7.28	0.826
Prior PCI	1.38	3.35	1.55	0.142
Prior CAD	44.19	67.33	46.09	**<0.0001**
Tobacco use	35.50	33.16	35.30	0.653
Alcohol Disorder	2.90	0	2.66	0.097
Family history of CAD	2.85	0	2.61	0.100
Hyperlipidemia	58.45	50.46	57.79	0.136
Peripheral vascular disease	9.16	11.69	9.37	0.425
Anemia	39.26	38.52	39.20	0.888
Atrial fibrillation	54.89	55.82	54.97	0.864
Primary Payer				0.066
Medicare	90.34	96.51	90.85	
Medicaid	1.68	2.11	1.71	
Private insurance	7.98	1.37	7.44	
Hospital characteristics				
Hospital bed size				**0.0003**
Small	14.31	1.51	13.26	
Medium	26.36	20.76	25.90	
Large	59.33	77.73	60.84	
Hospital teaching status^¶^				**0.0026**
Non-Teaching	18.88	6.36	17.86	
Teaching	81.12	93.64	82.14	
Admission type				**<0.0001**
Non elective	95.83	27.15	90.20	
Elective	4.17	72.85	9.80	
Admission day				0.0062
Weekdays	78.95	90.84	79.93	
Weekend	21.05	9.16	20.07	
Disposition				<0.0001
Home	61.05	81.93	62.76	
Facility/others	38.95	18.07	37.24	
Length of stay (days)	8.351 (±0.366)	6.614 (±1.217)	8.201 (±0.350)	0.176

Values are mean ± SD or %. *The p value comparing TAVR to no TAVR. ‡International Classification of Diseases-10th Revision codes were used to identify respective comorbidities as per Supplemental Table 2. The bed size cutoff points divided into small, medium, and large have been done so that approximately one-third of the hospitals in a given region, location, and teaching status combination would fall within each bed size category (https://www.hcup-us.ahrq.gov/db/vars/hosp_bedsize/nrdnote.jsp). ¶A hospital is considered to be a teaching hospital if it has an American Medical Association–approved residency program (https://www.hcup-us.ahrq.gov/db/vars/hosp_ur_teach/nrdnote.jsp.).

OSA = obstructive sleep apnea; COPD = chronic obstructive pulmonary disease; CKD = chronic kidney disease; CABG = coronary artery bypass graft; PCI = percutaneous coronary intervention; CAD = coronary artery disease;

## 3 Results

A total of 1127 patients were included in the final analysis, of which 92 (8.2%) underwent TAVR (Table 1**)**. A larger proportion of the cohort was male (62.3%) than female and the majority were older than 80 years (57.9%). The most common comorbidities were hypertension (88.5%), CKD stage 3 or more (52.4 %), hyperlipidemia (57.8 %), and AF (55.0%). Medicare was the most common insurance payer (90.9%), and patients were more likely to present to large (60.8%) or teaching (82.1%) hospitals. Weekday admissions (79.9%) were more common than weekend admissions and patients were more commonly discharged home (62.8%) than to other facilities.

Patients who underwent TAVR were more likely to have a history of coronary artery disease (67.33% vs. 44.19%, p < 0.0001) and less like to have a history of TIA or stroke (25.72% vs 14.12%, p = 0.013) as compared to patients who did not undergo TAVR. Patients who underwent TAVR were also more likely to have been admitted to large hospitals (77.73% vs 59.33%, p = 0.0003), admitted to teaching hospitals (93.64% vs 81.12%, p = 0.0026), admitted electively (72.85% vs 4.17%, p < 0.0001), and discharged home at time of disposition (81.93% vs 61.05%, p < 0.0001) as compared to patients who did not undergo TAVR. There was no statistically significant difference in length of stay in patients who underwent TAVR as compared to those who did not (6.6 days vs 8.4 days, p = 0.176).

**Fig. 2:**
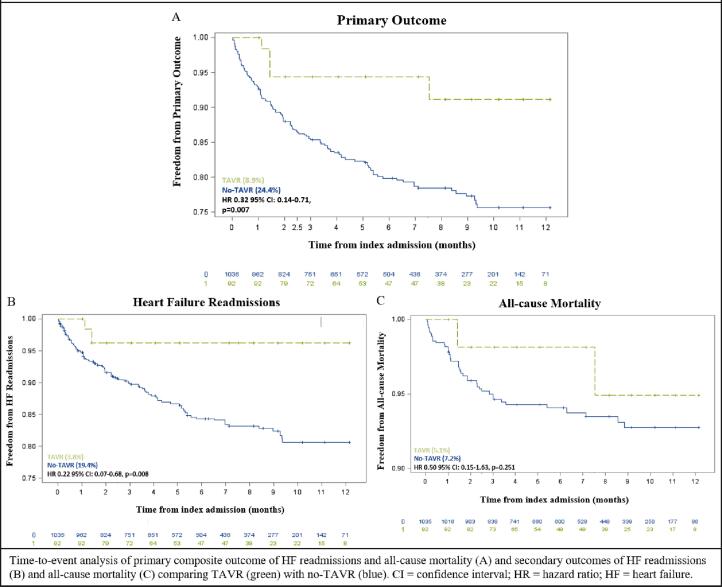
Kaplan-Meier Curves of Primary and Secondary Outcomes.

The composite primary outcome was lower in patients who received TAVR (8.9% vs 24.4%, HR 0.32; 95% CI 0.14–0.71, p = 0.007) as compared to those who did not receive TAVR (Table 2**,** Fig. 2). Among secondary outcomes, the incidence of admissions for acute decompensated heart failure was lower in patients who underwent TAVR as compared to those who did not (3.8% vs 19.4%, HR 0.22; 95% CI 0.07–0.68, p = 0.008) (Table 2**,** Fig. 2). There was no statistically significant difference in all-cause mortality in patients who underwent TAVR as compared to those who did not receive TAVR (5.1% vs 7.2%, HR 0.50; 95% CI 0.15–1.63, p = 0.251) (Table 2**,** Fig. 2).

**Table 2:** Primary and Secondary Outcomes by TAVR Status.

	No TAVR (n = 1035)	TAVR (n = 92)
Primary outcome		
Events (n)	197	6
Cumulative percentage (%)¶	24.4	8.9
HR‡ (95% CI, p-value) (unadjusted)	0.32(0.14–0.73, 0.006)	
HR (95% CI, p-value) (adjusted*)	0.32 (0.14–0.71, 0.007)	
Heart failure readmission		
Events (n)	147	3
Cumulative percentage (%)	19.4	3.8
HR (95% CI, p-value) (unadjusted)	0.23 (0.07–0.69, 0.009)	
HR (95% CI, p-value) (*adjusted)	0.22 (0.07–0.68, 0.008)	
All-cause mortality		
Events (n)	62	3
Cumulative percentage (%)	7.2	5.1
HR (95% CI, p-value) (unadjusted)	0.0.53 (0.16–1.71, 0.286)	
HR (95% CI, p-value) (adjusted)	0.50 (0.15–1.63, 0.251)	
All-cause readmission		
Events (n)	424	31
Cumulative percentage (%)	49.9	39.9
HR (95% CI, p-value) (unadjusted)	0.78 (0.54–1.12, 0.183)	
HR (95% CI, p-value) (adjusted)	0.76 (0.53–1.10, 0.145)	

HR = Hazard ratio.

¶ Cumulative percentages using Kaplan-Meier curve time-to-event analysis.

‡ A Cox proportional hazards regression model was used to generate HRs. Separate models were used for each outcome.

* All multivariate models are adjusted for age and gender.

In subgroup analysis of outcomes in patients who underwent TAVR, a lower rate of the primary outcome was noted in patients age < 80 years, male gender, CKD stage 3 or more, large hospital size, and teaching hospital status (**Supplementary Table 1)**. No significant difference in the primary outcome was noted in subgroups of patients age > 80 years, COPD, prior CAD, anemia, or small/medium bed size. (**Supplementary Table 1**).

## 4 Discussion

The relatively recent recognition of the coexistence of aortic stenosis in cardiac amyloidosis in addition to the development of novel therapies to address wild type and hereditary forms of cardiac amyloid have prompted the re-evaluation of approaches to the treatment of this disease. There is currently no consensus regarding the optimal management strategy in patients with CA and AS due to small, limited, and conflicting studies regarding outcomes after transcatheter and surgical aortic valve replacement [13], [14]. The objective of this study was to determine practice patterns of TAVR utilization as well as outcomes in patients with aortic stenosis and underlying cardiac amyloidosis.

In this retrospective cohort study, patients with CA and AS who underwent TAVR had a significantly lower rate of a composite outcome of all-cause mortality and heart-failure readmissions, driven primarily by a significantly lower rate of heart failure readmissions. There was no significant decrease in mortality associated with patients who underwent TAVR. Subgroup analysis of outcomes demonstrated patients age < 80 years, male gender, CKD stage 3 or more, presentation to large hospital size, and presentation to teaching hospital were associated with lower rate of the composite endpoint of heart failure readmission and mortality. Patients with AS and CA were elderly with comorbidities of hypertension, chronic kidney disease, and atrial fibrillation as would be expected in this population. Only 8% underwent TAVR, potentially due to concerns of pursuing TAVR in patients with limited life expectancy. The subset of CA patients who underwent TAVR were more likely to be aged < 80 years, suggesting potential selection of patients who are younger and less likely to be functionally limited. Notably, there were higher rates of coronary artery disease in patients who underwent TAVR as compared to those who did not.

The potential benefit of TAVR in CA is highlighted by results from the ATTRact-AS study, which involved 26 patients in the United Kingdom with both CA and severe AS and found a significant mortality benefit from TAVR in comparison to medical therapy [15]. These results contrast with our own analysis and previously cited data from other cohorts [13]. An explanation for this discrepancy may lie in the various distinctions between our cohort and the ATTRact-AS cohort. The ATTRact-AS study recruited patients who were referred for TAVR, after which all patients underwent ^99m^Tc-3,3-diphosphono-1,2-propanodicarboxylic acid (DPD) bone scintigraphy to look for evidence of ATTR CA. Patients with clinically active, previously diagnosed CA were not the primary target group. Furthermore, all patients fell into DPD grade 1 (31%) or grade 2 (69%) [15]. In addition, our follow up period was up to 1 year compared with up to 3 years in ATTRact-AS. Overall, both ATTRact-AS and our study showed an improvement in our primary outcomes and recommend considering TAVR in select CA patients with AS.

In our analysis, we were able to identify various factors that drove the reduction in our primary outcome in the TAVR group, including age < 80 years, male gender, CKD stage 3 or more, large hospital size, and teaching hospital status. Previous studies corroborate our findings that younger age and higher hospital TAVR volume (likely correlated with larger hospital size) are associated with lower heart failure readmissions and mortality[16], [17]. Interestingly, a previous *meta*-analysis of the short-term TAVR outcomes comparing teaching and non-teaching hospitals noted higher rates of acute kidney injury, hospital length of stay, and TAVR-related cost in teaching hospitals, however long-term mortality and heart failure readmissions rates were not assessed [18]. Large academic medical centers are likely to be better equipped to perform TAVR in CA patients due to the complexity of this patient population.

## 5 Limitations

First, this is an observational, retrospective analysis. Additionally, the groups compared in our analysis were not completely equivalent (prevalence of TIA or stroke, CAD, etc.) which may confound our results. Although 1127 patients were included in our analysis, only 92 patients (8.2%) underwent TAVR. Still, to our knowledge, we report the largest analysis of CA patients undergoing TAVR. Furthermore, our follow-up period was 1 year. Some components of our analysis may have reached statistical significance if this period was longer. Additionally, we were unable to reliably determine the severity of aortic stenosis in our cohort, but it was likely moderate to severe in most cases as mild disease is typically not noted at the time of medical coding. We were also unable to determine if a Heart Team approach was used in the evaluation of patients being considered for valve replacement. Finally, we were unable to account for the functional status or frailty of patients, which likely played a role in patient selection for TAVR and outcomes.

## 6 Conclusions

TAVR is associated with a significantly lower rate of a composite outcome of all-cause mortality and heart-failure in patients with CA and AS. CA patients who underwent TAVR were more likely to be younger and have a history of CAD as compared to those who did not. Subgroup analysis of outcomes demonstrated patients age < 80 years, male gender, CKD stage 3 or more, presentation to large hospital size, and presentation to teaching hospital were associated with lower rate of the composite endpoint of heart failure readmission and mortality. Future TAVR studies are necessary to understand the benefits of this intervention in the CA population.

## Funding

This study did not receive any financial support.

## Disclosures

Steven J. Filby, MD is a consultant for Boston Scientific. Guilherme F. Attizzani, MD is a consultant, proctor, and is on the advisory board of Medtronic and is a consultant for Abbott Vascular. The remaining authors have no disclosures to report.

## Declaration of Competing Interest

The authors declare that they have no known competing financial interests or personal relationships that could have appeared to influence the work reported in this paper.
