# Supplementary material for: Transcatheter aortic valve replacement in patients with aortic stenosis and cardiac amyloidosis
Source: Int J Cardiol Heart Vasc. 2022 Mar 21;40:101008. doi: 10.1016/j.ijcha.2022.101008 (PMC8938882; doi:10.1016/j.ijcha.2022.101008)
Supplement: Supplementary data 1 [file mmc1.docx]

| Supplementary Table 1: Subgroup Analysis | |  |  |  |  |
| --- | --- | --- | --- | --- | --- |
|  | n | HR | LL | UL | p-value |
| Age (Years) |  |  |  |  |  |
| < 80 | 474 | 0.12 | 0.02 | 0.67 | 0.015 |
| ≥80 | 653 | 0.58 | 0.23 | 1.48 | 0.256 |
| Gender |  |  |  |  |  |
| Male | 701 | 0.44 | 0.2 | 0.99 | 0.049 |
| Female | 425 | . | . | . | . |
| Comorbidities |  |  |  |  |  |
| COPD | 185 | 0.96 | 0.31 | 3.01 | 0.941 |
| CKD stage 3 or more | 591 | 0.8 | 0.15 | 0.96 | 0.04 |
| Prior CAD | 519 | 0.5 | 0.22 | 1.14 | 0.099 |
| Anemia | 442 | 0.36 | 0.12 | 1.12 | 0.078 |
| Hospital bed size |  |  |  |  |  |
| Small/Medium | 441 | 0.46 | 0.09 | 2.25 | 0.334 |
| Large | 686 | 0.28 | 0.11 | 0.71 | 0.007 |
| Hospital teaching status |  |  |  |  |  |
| Non-Teaching | 201 | . | . | . | . |
| Teaching | 926 | 0.34 | 0.15 | 0.76 | 0.009 |
| Abbreviations as in Table 1. | | | | | |

| **Supplementary Table 2: ICD-10 CM codes used to identify comorbidities** | |
| --- | --- |
|  | **ICD 10 CM codes** |
| **Comorbidities** | **Secondary diagnosis field** |
| OSA‡ | G47.33 |
| Obesity | E66, Z68.3, Z68.4 |
| Hypertension | I10, I11, I12, I13, I15, I16 |
| Diabetes | E08, E09, E10, E11, E13 |
| history of TIA or stroke^††^ | I69.3, Z86.73 |
| COPD^§^ | J41, J42, J43, J44, |
| CKD^‖^ stage 3 or more | N18.3, N18.4, N 18.5, N18.6, E08.2, E09.2, E10.2, E11.2, E13.2, I12, I13, |
| Prior CABG^**^ | I25.70, I25.71, I25.72, I25.73, I25.76, I25.79, I25.810, I25.812, T82.211, Z95.1 |
| Prior PCI^#^ | Z98.61 |
| Prior CAD^&^ | I25 |
| Tobacco use | F17, Z87.891, Z72.0, O99.33, T65.2 |
| Alcohol Disorder | F10, Z71.40, K29.2, G31.2, K85.2, K86.0, T51, I42.6, K70, G62.1 |
| Hyperlipidemia | E78.0, E78.1, E78.2, E78.4, E78.5 |
| Family history of CAD^&^ | Z82.49 |
| Peripheral vascular disease | E08.5, E09.5, E10.5, E11.5, E13.5, I73, T82.856, Z98.62, Z95.820 |
| Anemia | D50, E60, D61, D62, D63, D64, D46.0, D46.1, D46.2, D46.4, O99.0 |
| Atrial fibrillation | I48 |
| &: CAD: coronary artery disease  ‡: OSA: obstructive sleep apnea  ††: TIA: transient ischemic attack  §: COPD: chronic obstructive pulmonary disease  ‖: CKD: chronic kidney disease  **: CABG: coronary artery bypass graft  #: PCI: percutaneous coronary intervention | |
